# A novel monoclonal antibody against human thymic stromal lymphopoietin for the treatment of TSLP-mediated diseases

**DOI:** 10.3389/fimmu.2024.1442588

**Published:** 2024-12-11

**Authors:** Lihua Shi, Mingcan Yu, Ying Jin, Peng Chen, Guangmao Mu, Susan H. Tam, Minseon Cho, Mark Tornetta, Chao Han, Man-Cheong Fung, Mark L. Chiu, Di Zhang

**Affiliations:** ^1^ Tavotek Biotherapeutics, Inc., Lower Gwynedd Township, PA, United States; ^2^ Tavotek Biotherapeutics, Inc., Suzhou, Jiangsu, China

**Keywords:** TSLP antibody, allergy, antibody engineering, chronic inflammation, autoimmune disease

## Abstract

**Introduction:**

Thymic stromal lymphopoietin (TSLP) is a master regulator of allergic inflammation against pathogens at barrier surfaces of the lung, skin, and gut. However, aberrant TSLP activity is implicated in various allergic, chronic inflammation and autoimmune diseases and cancers. Biologics drugs neutralizing excess TSLP activity represented by tezepelumab have been approved for severe asthma and are being evaluated for the treatments of other TSLP-mediated diseases.

**Methods and results:**

In this study, we discovered and characterized a novel humanized anti-TSLP antibody TAVO101 with high binding affinity to human TSLP, which blocks TSLP binding to its receptor complexes on cell surface. TAVO101 showed potent neutralization of TSLP activities in the TSLP-driven STAT5 reporter assay and cell proliferation assay. Results from *ex vivo* studies showed that TAVO101 neutralized TSLP-mediated CCL17 release from primary human CD1c^+^ dendritic cells and proliferation of activated CD4^+^ T cells. In addition, TAVO101 showed strong efficacy in both TSLP/OVA-induced asthma and imiquimod induced psoriasis models in hTSLP/hTSLPR double knock-in mice. We further conducted Fc engineering to optimize TAVO101 antibody with reduced affinity to Fcγ receptors and C1q protein but with increased affinity to FcRn receptor for half-life extension.

**Discussion:**

By recognizing a different epitope, similarly potent neutralization of TSLP activities, and longer circulating half-life than tezepelumab, novel anti-TSLP antibody TAVO101 offers a potential best-in class therapeutics for various TSLP-mediated diseases.

## Introduction

The pro-inflammatory thymic stromal lymphopoietin (TSLP) is a member of the interleukin-2 (IL-2) family of cytokine and is a master regulator of allergic inflammation. As one of the epithelial cell derived cytokines or alarmins that are widely expressed and secreted in epithelial cells and epidermal keratinocytes of the lung, skin and gut upon allergen challenges (1, 2), TSLP acts on many different types of hematopoietic and non-hematopoietic cells that include dendritic cells, mast cells, basophils, eosinophils, monocytes, T cells, and B cells (3–6). TSLP promotes myeloid-derived dendritic cell maturation and proliferation. The activated dendritic cells can prime naïve T helper cells to adopt a Th2 phenotype by producing high concentrations of Th2 cytokines (7). This in turn can recruit eosinophilic and basophilic granulocytes and mast cells into the inflammatory tissue to secrete more inflammatory cytokines and chemokines for the establishment of allergic inflammation. Therefore, by coordinating both innate and adaptive immune response, TSLP is a key cytokine critical for mediating allergic Th2 inflammation at barrier surfaces against pathogens (8).

TSLP, a distant paralog of IL-7, has a four-helical bundle structure with six conserved cysteine residues and multiple sites for N-linked carbohydrate addition (3). TSLP signals through a heterodimeric receptor complex composed of the thymic stromal lymphopoietin receptor (TSLPR) and the IL-7R alpha-chain (IL-7Rα) (9). Human TSLP alone can bind to TSLPR by a long-range electrostatic attraction but has no affinity for IL-7Rα. The TSLP-TSLPR binary complex has enhanced binding affinity to IL-7Rα to form a ternary signaling complex (10). On binding to its cognate receptor complex, TSLP can activate multiple signal transduction pathways that includes activation of Janus kinases (JAKs), particularly JAK1 and JAK2 (11–13). Activated JAKs, in turn, regulate the activity of multiple Signal Transducer and Activator of Transcription (STAT) factors, including STAT5, STAT3 and STAT1. Besides JAK/STAT pathway, Src-related tyrosine kinase is involved in TSLP-induced cell proliferation since cell proliferation is blocked by the Src family inhibitor (14). In addition, TSLP protects against liver ischemia/reperfusion injury and hypoxic cell death via activation of the PI3K/Akt pathway and the activation of autophagy in hepatocytes (15).

While TSLP is a key regulator in mediating Th2 allergic inflammation, excessive TSLP activity in the airway epithelium can trigger and perpetuate atopic asthma, a common disorder characterized by tissue obstruction and remodeling in the airway, bronchial smooth muscle cell hyperreactivity to allergens and chronic bronchial inflammation (16, 17). Besides allergic diseases in the airway such as asthma, aberrant TSLP activity has been linked to allergic diseases of the skin and gut, including atopic dermatitis, allergic rhinitis, and food allergy (18–21). TSLP is also involved in chronic inflammatory diseases such as chronic obstructive pulmonary disease (COPD) and inflammatory bowel disease (IBD) (22, 23). More recently, TSLP is implicated in autoimmune diseases including psoriasis vulgaris and rheumatoid arthritis (24–27). In addition, more evidence are emerging implicating TSLP involvement in different types of cancers (28).

As the key driver in allergic diseases and other types of immune disorders, blocking dysfunctional TSLP activity can be used to treat these diseases. Tezepelumab (AMG157/MEDI9929) is a human anti-TSLP monoclonal antibody that specifically binds TSLP and blocks TSLP interaction with its cognate receptor complex (29). Two randomized, double-blind, placebo-controlled phase I studies revealed good pharmacokinetics, safety, and tolerability profiles for tezepelumab (30). A proof-of-concept study indicated that tezepelumab could attenuate allergen-induced airway responses in patients with mild atopic asthma (29). Upon subsequent clinical trials demonstrating efficacious treatment of patients with severe asthma, tezepelumab was FDA approved as an add-on maintenance treatment for adults and children aged 12 years and older with severe asthma not controlled by their current asthma medicine (31). However, tezepelumab did not show efficacy in the treatment of moderate to severe atopic dermatitis in clinical trials (32). Nonetheless, more clinical trials are ongoing to evaluate tezepelumab efficacies in other TSLP-mediated allergic, inflammatory, and autoimmune disorders that include severe chronic rhinosinusitis with nasal polyposis, chronic obstructive pulmonary disease, and chronic spontaneous urticaria.

As tezepelumab is the only TSLP antagonistic antibody approved so far for the treatment of TSLP-related diseases, there is a need for the development of additional anti-TSLP antibody with improved efficacies and pharmacokinetic profiles. Since allergen exposure is unpredictable, a prolonged treatment is required to obtain more potent sustained disease control in addition to improving convenience and patient compliance. In this study, we discovered and characterized a novel humanized anti-TSLP TAVO101 antibody with potent neutralization of TSLP activities in multiple *in vitro*, *ex vivo*, and *in vivo* studies. In addition, we engineered TAVO101 antibody to have an extended half-life and reduced engagement to Fcγ receptors for better safety profile. In our Phase 1 clinical trial, TAVO101 was safe, well-tolerated and exhibited good pharmacokinetics and immunogenicity profiles (33). Overall, our data supported further development of TAVO101 in the clinic as a potential best-in-class treatment of various TSLP-mediated diseases.

## Materials and methods

### Antibody expression and purification

Recombinant antibodies were produced by cloning the antibody genes into an expression vector followed by transfecting the expression constructs into a host cell line to express the antibody for purification. Gene fragments encoding antibody heavy chain (HC) and light chain (LC) were synthesized by Genewiz (South Plainfield, NJ) or IDT (Coralville, IA) and cloned into pcDNA3.4 expression vector (ThermoFisher Scientific, San Jose, CA). Antibody heavy chain and light chain constructs (1:3 HC to LC molar ratio) were co-transfected into Expi293F cells by Expifectmine293 transfection kit (ThermoFisher Scientific, San Jose, CA). Cells were incubated for 5 days at 37°C and the supernatant was harvested and purified by affinity chromatography over MabSelect SuRe column followed by a desalting column (Cytiva, Marlborough, MA). Protein concentration was determined by Nanodrop (ThermoFisher Scientific, San Jose, CA) with UV absorbance at 280 nm. The tezepelumab analogue antibody (hitherto referred to just as tezepelumab) was generated in-house based on heavy chain and light chain sequences for tezepelumab deposited in the protein Data Bank (5J13).

### ELISA binding assay

Recombinant human, mouse, and cynomolgus monkey TSLP were obtained from AcroBiosystems (Newark, DE). Briefly, Maxisorp 96-well plate was coated with recombinant TSLP at the 1 μg/mL concentration in DPBS pH 7.2 and incubated overnight at 4°C. After blocking with blocking buffer, serial 3-fold dilutions of test antibodies starting at 1 μg/mL were applied to the plate and incubated for 2 hours at room temperature by shaking. After washing the plates four times with washing buffer, HRP-conjugated anti-human IgG secondary antibody (Jackson ImmunoResearch Laboratories, West Grove, PA) were added for the detection. Plate was washed four times with wash buffer then TMB (3,3´,5,5´-tetramethylbenzidine) substrate (BioLegend, San Diego, CA) was added to the plate for color development. Optical densities were determined with the SpectraMax i3X plate reader (Molecular Devices, Sunnyvale, CA) at 450 nm wavelength. Data was plotted as absorbance at 450 nm versus the logarithm of antibody concentration. Nonlinear regression analysis was performed by GraphPad Prism 10.2.3 (GraphPad Software, La Jolla, CA) and EC_50_ values of binding were calculated.

### Blocking TSLP binding to its cell surface receptor complex

HEK293T cells were co-transfected with constructs expressing human IL-7Rα and TSLPR using the lipofectamine 3000 according to the manufacturer’s instructions. Two days after transfection, serial 3-fold dilutions of test antibodies starting at 10 μg/mL along with 5 ng/mL of biotinylated human TSLP (Acro biosystems, Newark Delaware) were applied to the cells and incubated for 2 hours. After washing the cells with staining buffer, PE-conjugated Streptavidin (BioLegend, San Diego, CA) were applied to the cells. The staining of biotinylated TSLP bound on the surface of transfected cells were evaluated by flow cytometry using MACSQuant Analyzer 10 (Miltenyi Biotec, Bergisch Gladbach, Germany). The mean fluorescence intensity (MFI) values were obtained and plotted against the logarithm of testing antibody concentrations. Nonlinear regression analysis was performed by GraphPad Prism 10.2.3 (GraphPad Software, La Jolla, CA) and IC_50_ values of blocking TSLP binding to its cell surface receptor complex were calculated.

### Bio-layer interferometry epitope binning assay

Label-free BLI was used to assess kinetic binding of TSLP by anti-TSLP antibodies using the Gator instrument (Gator Bio, Palo Alto, CA). The binding kinetics for TAVO101 and tezepelumab were obtained by using anti-Fc biosensor probes. Each antibody was incubated at 2 microgram/mL with the anti-Fc biosensor probes which reached a signal equilibrium of 1nm. The analyte, TSLP, was applied at concentrations ranging from 3 microgram/mL to 0.3 microgram/mL. Association and dissociation runs were set for 600 and 900 seconds, respectively. Kinetic constants were established by Gator analysis software set for 1:1 model binding and applying the partial universal curve fit. Sequential competition assay applied biotinylated TSLP (AcroBiosystems, Newark, DE) diluted at 1 µg/ml and streptavidin-coated biosensor probes. The first analyte, either TAVO101 or tezepelumab, at 4 microgram/mL was incubated with the TSLP-bound biosensor until a signal of equilibrium was reached, usually within 600 seconds. The sensor was then dipped into kinetics buffer for 10 seconds. The second analyte, either tezepelumab or TAVO101, were incubated for 600 seconds.

### TSLP-mediated STAT5 reporter gene assay

pGL4.52 reporter plasmid (Promega, Madison MI) carries five copies of STAT5-responsive motifs that drive the transcription and expression of the luciferase reporter gene luc2P. HEK293T cells were plated at a density of 10000 cells/well into 96 well white flat bottom tissue culture-treated microplate. The next day, cells were co-transfected with constructs expressing IL7Rα and TSLPR, and pGL4.52 STAT5 luciferase reporter gene construct using the lipofectamine 3000 according to the manufacturer’s instructions. One day post-transfection, the transfected cells were applied with serial 3-fold dilutions of human TSLP protein (R&D system) starting at 100 ng/mL for 24 hours. The luciferase activity was determined using the ONE-Glo™ EX Luciferase Assay kit (Promega, Madison MI) according to the manufacturer’s instruction. The fluorescence data was collected using the SpectraMax i3X plate reader (Molecular Devices, Sunnyvale, CA) and plotted against the logarithm of TSLP concentrations. Nonlinear regression analysis was performed by GraphPad Prism 10.2.3 and the EC_50_ value of TSLP was calculated.

To evaluate the activities of anti-TSLP antibodies in neutralizing TSLP-mediated STAT5 reporter gene activation, the cells were treated with serial 3-fold dilutions of antibodies starting at 10 μg/mL along with 3ng/mL TSLP and the luciferase reporter gene expression were quantitated as described above. The percentages of TSLP activity were normalized to the maximum TSLP-driven activity in the case without the addition of antibody and plotted against the logarithm of testing antibody concentrations. Nonlinear regression analysis was performed by GraphPad Prism 10.2.3 and IC_50_ values of functional neutralization were calculated.

### TSLP-driven cell proliferation assay

BaF3 mouse pro-B cells (ATCC, Manassas, VA) co-transfected with human IL-7Rα and TSLPR expression constructs were maintained in RPMI 1640 medium supplemented with 10% FBS and 50 μM 2-mercaptoethanol. The transfected BaF3 Cells were washed three times with RPMI medium and seeded into the 96 well plates at a density of 5000 cells per well. Then the cells were applied with serial 3-fold dilutions of human TSLP (R&D system) at starting concentration of 15 ng/ml and incubated in a 37°C, 5% CO_2_ humidified incubator for 2 days. 10 μL/well of a 0.1 mg/mL resazurin solution was added and incubated for another 16-20 hours. At the end of the incubation, the fluorescent intensity was measured with an excitation wavelength set at 544 nm and an emission wavelength set at 590 nm. The fluorescence data was collected using SoftMax Pro 7 (Molecular Devices, San Jose, CA) and plotted against the logarithm of TSLP concentrations. Nonlinear regression analysis was performed by GraphPad Prism 10.2.3 and the EC_50_ value of TSLP was calculated.

For the neutralization of TSLP-driven cell proliferation by anti-TSLP antibodies, the transfected cells were treated with 0.5 ng/mL hTSLP and serial 3-fold dilutions of antibodies starting at 1 μg/mL and the proliferation of transfected cells were quantitated as described above. The fluorescence signals reflecting cell proliferation were plotted against the logarithm of testing antibody concentrations. Nonlinear regression analysis was performed by GraphPad Prism 10.2.3 and IC_50_ values of functional neutralization were calculated.

### TSLP-mediated CCL17 release from activated dendritic cell assay

CD1c^+^ dendritic cells (DCs) were isolated from peripheral blood mononuclear cells (PBMC) (Hemacare, Northridge, CA) from healthy donors with the CD1c^+^ Dendritic Cell Isolation Kit (Miltenyi Biotec, Bergisch Gladbach, Germany) according to the manufacturer’s instructions. CD1c^+^ DCs were cultured in RPMI medium supplemented with 10% fetal calf serum (ThermoFisher Scientific, San Jose, CA). The isolated CD1c^+^ dendritic cells were treated with human TSLP at 15 ng/mL and the testing antibodies at 1 μg/mL for 2 days. The cell supernatants were collected and the TSLP-driven release of CCL17 protein was quantitated using the human CCL17/TARC kit (R&D Systems, Minneapolis, MN).

### TSLP-driven proliferation of activated human CD4^+^ T cell assay

Human CD4^+^ T cells were isolated from PBMC (Hemacare, Northridge, CA) from healthy donors with the MojoSort Human CD4 T cell isolation kit (BioLegend, San Diego, CA) according to the manufacturer’s instructions. Purified T cells were cultured in RPMI medium supplemented with 10% fetal bovine serum and labelled by 5 μM Cell Proliferation Dye eFluor 450 (ThermoFisher Scientific, San Jose, CA). The labelled CD4^+^ T cells at 2 x 10^5^ cells/well were activated by anti-CD3 (OKT3) antibody coated on plate at 5 μg/mL and treated with or without 50 ng/mL human TSLP and testing antibodies for six days. The labelled CD4^+^ T cells were then analyzed by flow cytometry using MACSQuant Analyzer 10 (Miltenyi Biotec, Bergisch Gladbach, Germany). The fractions of proliferated CD4^+^ T cells stained with serially diluted eFluor 450 dye were quantitated.

### FcγRI (CD64), FcγRIIIA (CD16a) and C1q binding assay

100 nM of biotinylated recombinant human FcγRI (CD64) or FcγRIIIA (CD16a) (AcroBiosystems, Newark, DE) were coated on 96-well plate pre-coated with streptavidin (Sigma, St. Louis, MO). Testing antibodies were added to the assay well and incubated for one hour at room temperature by shaking. After washing the plates four times, the binding of the antibodies to immobilized FcγRI (CD64) or FcγRIIIA (CD16a) were detected by an HRP-conjugated anti-Human IgG secondary antibody (Jackson ImmunoResearch Laboratories, West Grove, PA) and color was developed by adding TMB substrate (Abcam, Cambridge, UK). The absorbance data was obtained at 450 nm and plotted versus the logarithm of antibody concentrations. Nonlinear regression analysis was performed by GraphPad Prism 10.2.3.

To detect the binding to C1q protein, serially diluted testing antibodies were coated on 96-well plate overnight. 2 μg/mL C1q protein (Quidel, San Diego, CA) conjugated with biotin was added to the assay well and incubated for two hours at room temperature by gentle shaking. After washing the plates four times, the binding of C1q by testing antibodies were detected by HRP-conjugated streptavidin (ThermoFisher Scientific, San Jose, CA) and color was developed by adding TMB substrate (Abcam, Cambridge, UK). The absorbance data was obtained at 450 nm and plotted versus the logarithm of antibody concentrations. Nonlinear regression analysis was performed by GraphPad Prism 10.2.3.

### FcRn binding assay

The FcRn binding assay was carried out on Maxisorp 96-well plate by coating 1 μg/mL of recombinant mouse FcRn (R&D systems, Minneapolis, MN) in DPBS pH7.2 and incubating overnight at 4°C. After washing and blocking the plate with the blocking buffer containing 0.05 M MES [2-(*N*-morpholino) ethanesulfonic acid], 1% (v/v) bovine serum albumin (BSA), 0.02% (v/v) Tween-20, pH 6.0, testing antibodies were added to the wells and incubated for 1 hour at room temperature by shaking. After washing the plates four times with washing buffer, HRP-conjugated anti-Human IgG secondary antibody (Jackson ImmunoResearch Laboratories, West Grove, PA) were added for the detection. Plate was washed four times with wash buffer, TMB substrate (BioLegend, San Diego, CA) was added to the plate for color development. Optical densities were determined with the SpectraMax i3X plate reader (Molecular Devices, Sunnyvale, CA) at 450 nm wavelength. Data was plotted as the absorbance at 450 nm versus logarithm of antibody concentrations. Nonlinear regression analysis was performed by GraphPad Prism 10.2.3 and EC_50_ values of binding were calculated.

### Pharmacokinetic study

TAVO101_IgG1-AALS antibody was evaluated in a cynomolgus monkey PK model for 5 weeks to determine whether the M428L/N434S mutations could extend antibody circulating half-life. TAVO101_IgG1-AALS antibody was administered as a single intravenous infusion at 4 mg/kg into a male naïve cynomolgus monkey. Plasma samples were collected at pre-dose, and at 1h, 2h, and on days 2, 5, 9, 12, 16, 21, 24, 26, 29, 32, 35 post-doses. Human IgG in plasma samples were quantitated by a standard ELISA method, in which goat anti-human IgG was the capture antibody and HRP conjugated goat anti-human IgG (H+L) was the detection antibody. Non-compartmental analysis was conducted for estimation of PK parameters using Phoenix WinNonlin 6.4 software.

### TSLP/OVA-induced asthma model using hTSLP/hTSLPR humanized mice

The allergic asthma mouse study was performed using hTSLP/hTSLPR double knock-in mice developed by Biocytogen Pharmaceuticals (Beijing, China). These transgenic mice have human *TSLP* and part of human *TSLPR* genes engineered into the genome of C57BL/6 mice to replace their murine counterparts to express full length human TSLP protein and a chimeric TSLPR protein with extracellular and transmembrane region of human TSLPR fused with cytoplasmic region of mouse TSLPR. These mice were randomly allocated into 6 groups (groups G1 to G6) based on their body weights and dosed with test articles according to dosing regimen specified. G1 is the blank control group in which 4 mice were treated with 40 μL PBS by intranasal administration every two days. Eight mice were allocated to each group of the G2 to G6 groups and the allergic asthma was induced and established by the administration to these mice with 40 μL PBS containing 10 μg OVA (ovalbumin) and 1 μg human TSLP intranasally every other day for two weeks. Due to the novelty of this transgenic mouse model without prior studies regarding the choice of dosage levels for reference, for drug efficacy testing, on Day -1 and Day 6, G3 to G5 group mice were dosed with TAVO101 at 1 mg/kg, 3 mg/kg, and 10 mg/kg respectively via intraperitoneal injection. Besides, due to resource constraints, single dosing was chosen for negative and positive control groups, with G2 group mice were dosed with 10 mg/kg Isotype control antibody as a negative control and G6 group mice were dosed with 3 mg/kg tezepelumab as a positive control. The study was terminated on Day 14 and bronchoalveolar lavage fluid (BALF) samples and lung tissues were collected for ELISA, flow cytometry and lung histopathology analysis.

At the end of the experiment, blood was collected from all animals and total IgE concentrations in the serum were determined by ELISA-based quantitation assay as per the manufacturer’s guideline. The middle and lower lobes of right lung tissues were processed to measure the levels of mouse IL-4, IL-5, IL-13 and CCL17 cytokines by ELISA-based quantitation assays. Bronchoalveolar lavage fluid from animals of all test groups were collected and the isolated cells underwent flow cytometry analysis. Total cell counts of mCD45^+^ leukocytes, mCD45^+^mSiglec-F^+^mCD11c^-^ eosinophils, mCD45^+^mSiglec-F^+^mCD11c^+^ alveolar macrophages, and mCD45^+^mSiglec-F^-^mCD11c^-^mCD11b^+^mLy6G^+^ neutrophils were determined. The upper lobes of right lung tissues were collected for histopathological analysis by hematoxylin and eosin (H&E) staining and Periodic Acid Schiff (PAS) staining. The pathological assessment includes scoring for inflammatory cell infiltration around blood vessels and bronchioles and scoring for eosinophil infiltration according to an asthma model scoring system (**Supplementary Table 1**) The PAS positive areas were also analyzed to reflect the presence of mucus and goblet cells in bronchioles. Results were represented by means and the standard error (Mean ± SEM). Data were analyzed by One-way ANOVA with Dunnett’s multiple comparisons test (* p<0.05, ** p<0.01, *** p<0.001, **** p<0.0001).

### Imiquimod induced psoriasis model using hTSLP/hTSLPR humanized mice

The imiquimod induced psoriasis mouse study was also performed using hTSLP/hTSLPR double knock-in mice developed by Biocytogen. Mice were randomly allocated into four groups (groups G1 to G4) based on their body weights. On study Day 1, mice back skins were depilated with an electric clipper under 3-5% isoflurane anesthesia. Throughout the nine-day study period, mice in the G2, G3 and G4 groups received a topical application of 62.5 mg/mouse Aldara cream containing 5% imiquimod (IMQ) on the exposed skin of the back every day, while mice in the G1 group received topical application of Vaseline in the same area as a naïve mice control group. Mice were dosed with test articles according to the dosing regimen specified. G2 group mice did not receive antibody treatment while tezepelumab and TAVO101 were administered to the G3 and G4 group mice respectively via intraperitoneal injection with the dose at 10 mg/kg on Day 1 followed by four additional doses at 3 mg/kg every other day (10/3/3/3/3 dosing).

Throughout the study, the average skin thickness was calculated daily from two measurements distal to each other, and the percentage of skin thickness changes was calculated and scored accordingly. Skin lesion redness and scaling were also scored daily as follows: 0, none; 1, mild; 2, moderate; 3, marked; 4, severe. A total PASI (Psoriasis Area and Severity Index) was then calculated as the sum of thickness, redness and scaling scores. At the end of the study, the skin lesion samples were collected for the quantitation of key inflammatory cytokine levels, including human TSLP, mouse IL-6, TNFα and IL-1β, by ELISA or Meso Scale Discovery (MSD). The skin lesion samples were also collected for the measurement of epithelium thickness and histopathological analysis by hematoxylin and eosin (H&E) staining. Results were represented by means and the standard error (Mean ± SEM). Data were analyzed by One-way ANOVA with Dunnett’s multiple comparisons test (* p<0.05, ** p<0.01, *** p<0.001, **** p<0.0001).

## Results

### Identification of a novel anti-human TSLP antibody TAVO101

**Figure 1:**
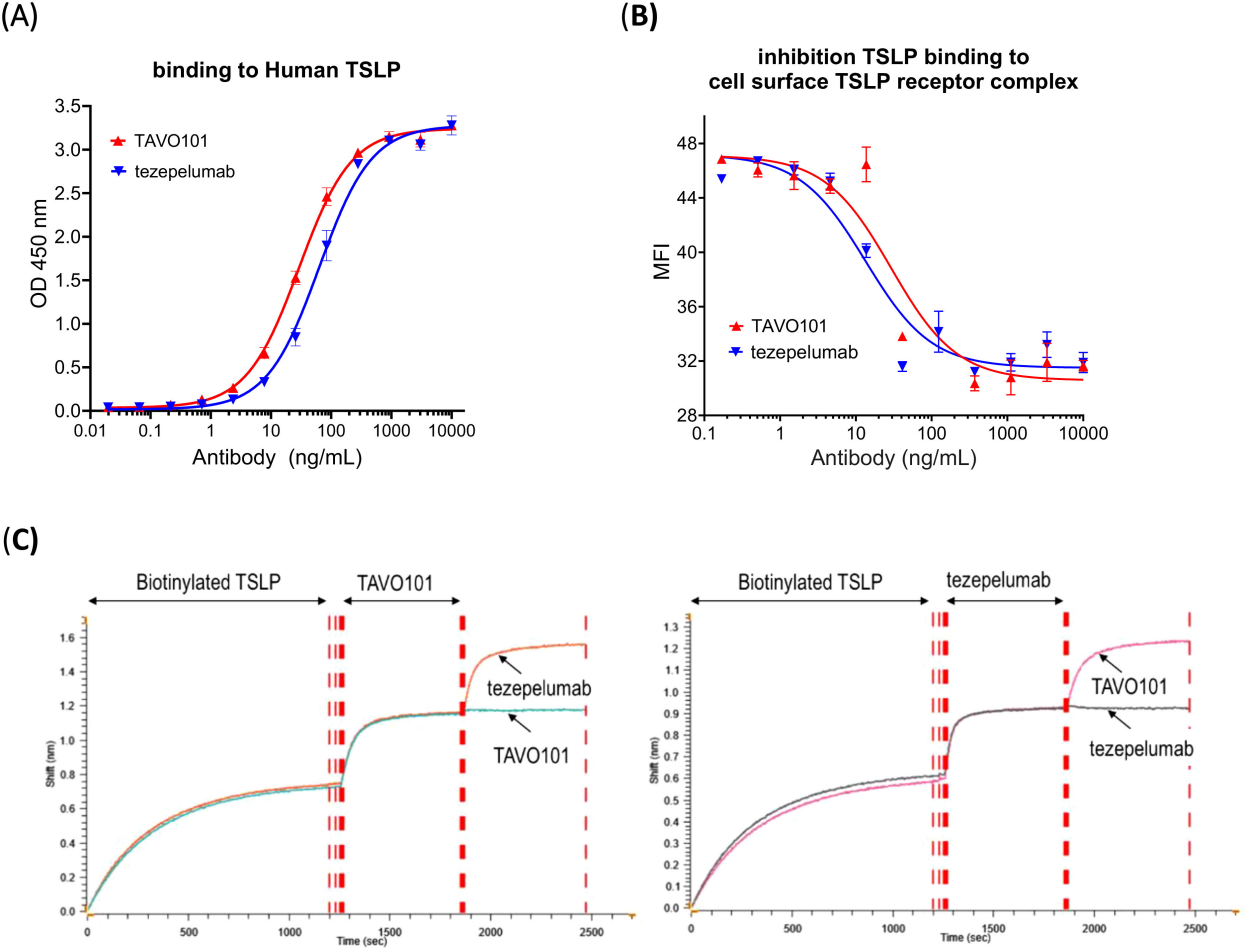
TSLP binding and epitope competition studies for TAVO101 and tezepelumab. **(A)**. Increasing concentrations of TAVO101 and tezepelumab were assessed for their binding to immobilized human TSLP. OD at 450 nm were plotted against the concentrations of test antibodies (Data expressed as mean ± SEM, n=2). **(B)**. Increasing concentrations of TAVO101 and tezepelumab were assessed for their inhibition on the binding of human TSLP to its receptor complex expressed on the surface of HEK293T cells transfected with IL7Rα and TSLPR by flow cytometry. Mean fluorescence intensities (MFI) representing TSLP binding were plotted against the concentrations of test antibodies (Data expressed as mean ± SEM, n=3). **(C)**. Epitope competition binding assays for TAVO101 and tezepelumab by gator Bio-layer Interferometry. After loading the biosensor with biotinylated TSLP during the first binding phase, either TAVO101 (left panel) or tezepelumab (right panel) was added until the binding to the TSLP on the biosensor was saturated during the second phase. In the third binding phase, either tezepelumab or TAVO101 was then added to monitor kinetic binding to the TSLP on the biosensor. Shifts in interference reflecting the binding of test antibodies to TSLP were plotted against time (in second) during the three phases of binding. In each assay, triplicate binding profiles for the competition antibody addition and a single binding profile for the same antibody addition during the third binding phase were shown.

A monoclonal mouse anti-human TSLP antibody was humanized by grafting mouse CDRs onto human germline IgG1 scaffolds with a few key mouse residues preserved by back mutations to achieve a higher stability and better expression while minimizing potential immunogenicity. An ELISA-based binding assay was employed to evaluate the humanized antibody, designated as TAVO101, binding to recombinant human TSLP antigen coated on ELISA plate. TAVO101 showed potent binding to human TSLP with EC_50_ of 29.06 ng/mL (0.19 nM), which was similar to reference antibody tezepelumab with EC_50_ of 63.12 ng/mL (0.42 nM) (**Figure 1A**). The binding of TAVO101 to cynomolgus monkey and mouse TSLP were also evaluated in similar ELISA binding assays by coating the plates with cynomolgus monkey and mouse TSLP antigens respectively. TAVO101 did not bind cynomolgus monkey TSLP while tezepelumab did bind (**Supplementary Figure 1A**). Neither TAVO101 nor tezepelumab bound to mouse TSLP while a control anti-mouse TSLP antibody bound (**Supplementary Figure 1B**).

The potencies of TAVO101 and tezepelumab in blocking TSLP binding to the human TSLP receptor complex composed of IL-7Rα and TSLPR expressed on cell surface of transfected HEK293T were assessed by flow cytometry assay. Both TAVO101 and tezepelumab concentration-dependently inhibited human TSLP binding to its receptor complex composed with human IL-7Rα and TSLPR (**Figure 1B**). TAVO101 and tezepelumab showed similar potency in the inhibition of human TSLP binding to its cell surface receptor complex with IC_50_ values of 29.13 ng/mL (0.2 nM) and 13.43 ng/mL (0.09 nM), respectively. By blocking TSLP binding to its cognate cell surface receptor, TAVO101 may functionally inhibit TSLP-mediated signal transduction.

### TAVO101 recognized a different epitope on TSLP from tezepelumab

The human TSLP binding affinities for TAVO101 and tezepelumab were also evaluated by BLI method of protein kinetic interactions. Using 3 concentrations of TSLP as the analyte both the kinetics of association and dissociation were monitored. The kinetic binding analysis determined similar binding rates of TAVO101 with an average K_D_ value of 0.85 nM (n=4) and tezepelumab with an average K_D_ value of 1.86 nM (n=4).

Competition binding assays using a three-phase BLI method assessed whether TAVO101 and tezepelumab shared similar binding epitopes on human TSLP. During the first binding phase, the biosensor was loaded with biotinylated TSLP. In the second phase, TAVO101 was added until the binding to the TSLP on the biosensor was saturated. In the third binding phase, either tezepelumab or TAVO101 was then added to monitor kinetic binding to the TSLP that was already bound by TAVO101 on the biosensor. Tezepelumab, but not TAVO101, bound to TSLP on the biosensor that was already saturated with TAVO101, indicating each antibody bound different epitopes (**Figure 1C**, left panel). The results were confirmed by switching around the second and third additions of the antibodies for the binding to TSLP. In reverse, when TSLP on the biosensor was saturated by the binding of tezepelumab, TAVO101 could still bind to TSLP while the additional tezepelumab could not bind (**Figure 1C**, right panel). These competition binding assay data indicated that TAVO101 and tezepelumab recognized different epitopes on human TSLP.

### Neutralization of TSLP activity by TAVO101

**Figure 2:**
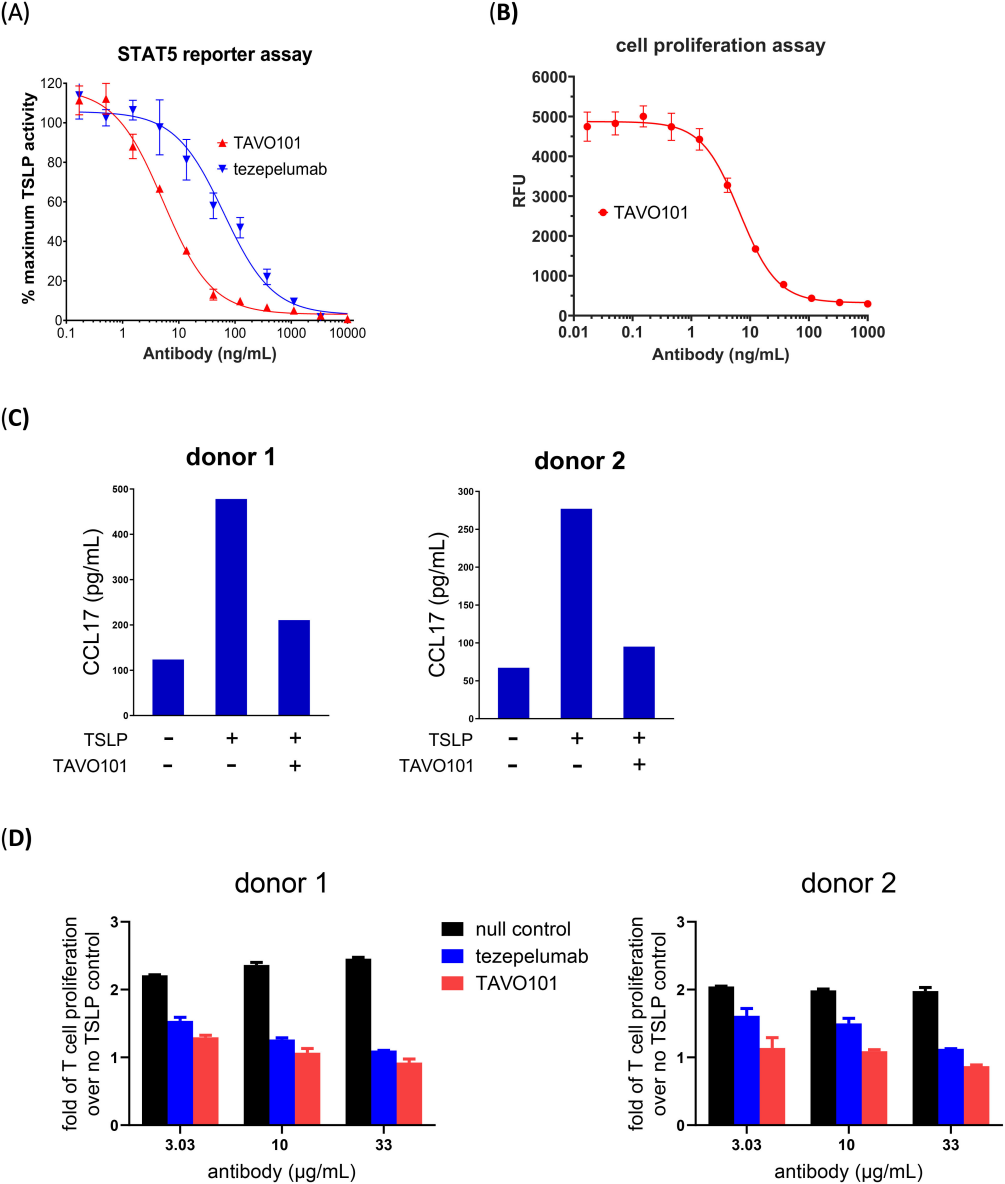
Neutralization of TSLP activity by TAVO101 and tezepelumab in functional potency assays. **(A)**. Neutralization of human TSLP-driven STAT5 reporter gene activation by TAVO101 and tezepelumab. Increasing amounts of TAVO101 or tezepelumab along with 3 ng/mL recombinant human TSLP were applied to HEK293T cells transfected with human TSLP receptor complex and a STAT5-responsive luciferase reporter gene and reporter gene expression was quantitated. The percentages of TSLP activity normalized to the maximum activity driven by 3 ng/mL human TSLP were plotted against the concentrations of testing antibodies (Data expressed as mean ± SEM, n=3). **(B)**. Neutralization of human TSLP-driven proliferation of BaF3 cells transfected with human TSLP receptor complex by TAVO101. Increasing amounts of TAVO101 along with 0.5 ng/mL recombinant human TSLP were applied to the transfected cells and cell proliferation was quantitated. Luminescence signals reflecting cell proliferation were plotted against the concentrations of TAVO101 (Data expressed as mean ± SEM, n=3). **(C)**. Neutralization of TSLP-driven CCL17 release from activated dendritic cells by TAVO101. CD1c^+^ blood dendritic cells isolated from the PBMC of two healthy donors were treated with 15 ng/mL human TSLP with or without 1 μg/mL TAVO101. The CCL17 releases from the activated dendritic cells were quantitated. The CCL17 levels were plotted against the testing antibodies in the bar graphs as shown. **(D)**. Neutralization of TSLP-driven proliferation of activated human CD4^+^ T cells. Human CD4^+^ T cells isolated from the PBMC of two healthy donors were labelled by Cell Proliferation Dye eFluor 450, activated by plate bound anti-CD3 antibody and treated with 50 ng/mL human TSLP with or without null control antibody, tezepelumab or TAVO101 at the indicated concentrations. The fraction of proliferated human CD4^+^ T cells were quantitated by flow cytometry as cells stained with diluted dyes. The fold of T cell proliferation over that without TSLP treatment were plotted against the testing antibodies in the bar graphs as shown. (Data expressed as mean ± SEM, n=2).

A TSLP-driven STAT5 reporter gene assay assessed the functional neutralization of TSLP activity by TAVO101 and tezepelumab. STAT5 activation is a downstream event that occurs after TSLP binds to and activates its receptor complex (TSLPR: IL-7Rα). In this assay, TSLPR and IL-7Rα expression constructs along with a STAT5-responsive luciferase reporter gene construct were transiently transfected into HEK293T cells. One day after transfection, recombinant human TSLP was applied to the cells and TSLP-driven luciferase reporter gene expression was quantitated 24 hours later. It was observed that human TSLP could concentration-dependently drive reporter gene expression with EC_50_ value of 1.1 ng/mL (**Supplementary Figure 2A**). The neutralization activity of the anti-TSLP antibodies were assessed by titrating varying concentrations of TAVO101 or tezepelumab with 3 ng/mL human TSLP onto HEK293T cells co-transfected with IL-7Rα, TSLPR, and STAT5-responsive luciferase reporter gene. TAVO101 demonstrated concentration-dependent neutralization of human TSLP from stimulating reporter gene expression with an IC_50_ value of 5.27 ng/mL (35.13 pM) while tezepelumab showed an IC_50_ value of 63.23 ng/mL (421.53 pM); about 12-fold less potent than TAVO101 (**Figure 2A**).

In addition, a TSLP-mediated cell proliferation assay evaluated the functional neutralization activity of TAVO101. In this assay, TSLPR and IL-7Rα were co-transfected into BaF3 mouse pro-B cells. Recombinant human TSLP was added to the transfected cells and cell proliferation was quantitated with a Resazurin cell viability assay two days later. The recombinant human TSLP stimulated the proliferation of the BaF3 cells expressing TSLP receptor complexes in a concentration-dependent manner with EC_50_ value of 0.17 ng/mL (11.3 pM) (**Supplementary Figure 2B**). To assess the functional activity of TAVO101, increasing concentrations of TAVO101 antibody along with 0.5 ng/mL human TSLP were applied with BaF3 cells co-transfected with IL-7Rα and TSLPR. The anti-TSLP antibody TAVO101 neutralized TSLP activity in stimulating transfected BaF3 cell proliferation with IC_50_ value of 6.6 ng/mL (44 pM) (**Figure 2B**).

TSLP can stimulate the activation of primary human CD1c^+^ dendritic cells with the increased secretion of CCL17, a chemokine which is involved in many allergy and inflammation reactions (34). An *ex vivo* assay evaluated TAVO101 neutralization of TSLP-driven CCL17 release from activated dendritic cells (10). CD1c^+^ dendritic cells were isolated from donor PBMC and incubated with recombinant human TSLP alone, or in the presence of TAVO101. The TSLP-induced release of CCL17 from dendritic cells was quantitated by ELISA. Fifteen ng/mL recombinant human TSLP stimulated enhanced CCL17 release from activated dendritic cells after 48 hours of incubation (**Figure 2C**). TAVO101 inhibited TSLP-driven CCL17 release from activated dendritic cells when added at 1 μg/mL along with TSLP. **Figure 2C** showed results from assays with PBMC isolated from two different donors.

TSLP can also directly stimulate the proliferation of activated human CD4^+^ T cells (35). An *ex vivo* assay evaluated TAVO101 neutralization of TSLP-driven proliferation of activated CD4^+^ T cells. Human CD4^+^ T cells isolated from donor PBMC were labelled with Cell Proliferation Dye eFluor 450 and activated by plate bound anti-CD3 antibody with or without 50 ng/mL TSLP for six days. The TSLP-driven proliferation of activated CD4^+^ T cells in terms of serial dilution of eFluor 450 signals were evaluated by flow cytometry. In these experiments, human TSLP enhanced the proliferation of activated human CD4^+^ T cells (**Supplementary Figure 2C**). When anti-TSLP antibodies were incubated with the activated CD4^+^ T cells along with TSLP, both TAVO101 and tezepelumab concentration-dependently inhibited TSLP-driven proliferation of activated CD4^+^ T cells. TAVO101 showed a trend of slightly better efficacy (albeit not statistically significant) in neutralizing TSLP activity than tezepelumab in these assays. **Figure 2D** showed the results from assays with PBMC isolated from two different donors. In summary, these *in vitro* and *ex vivo* studies demonstrated how TAVO101 effectively neutralized TSLP-driven signal transduction pathways leading to the activation of allergic inflammation responses in target cells.

### Efficacy of TAVO101 in an TSLP/OVA-induced asthma model

**Figure 3:**
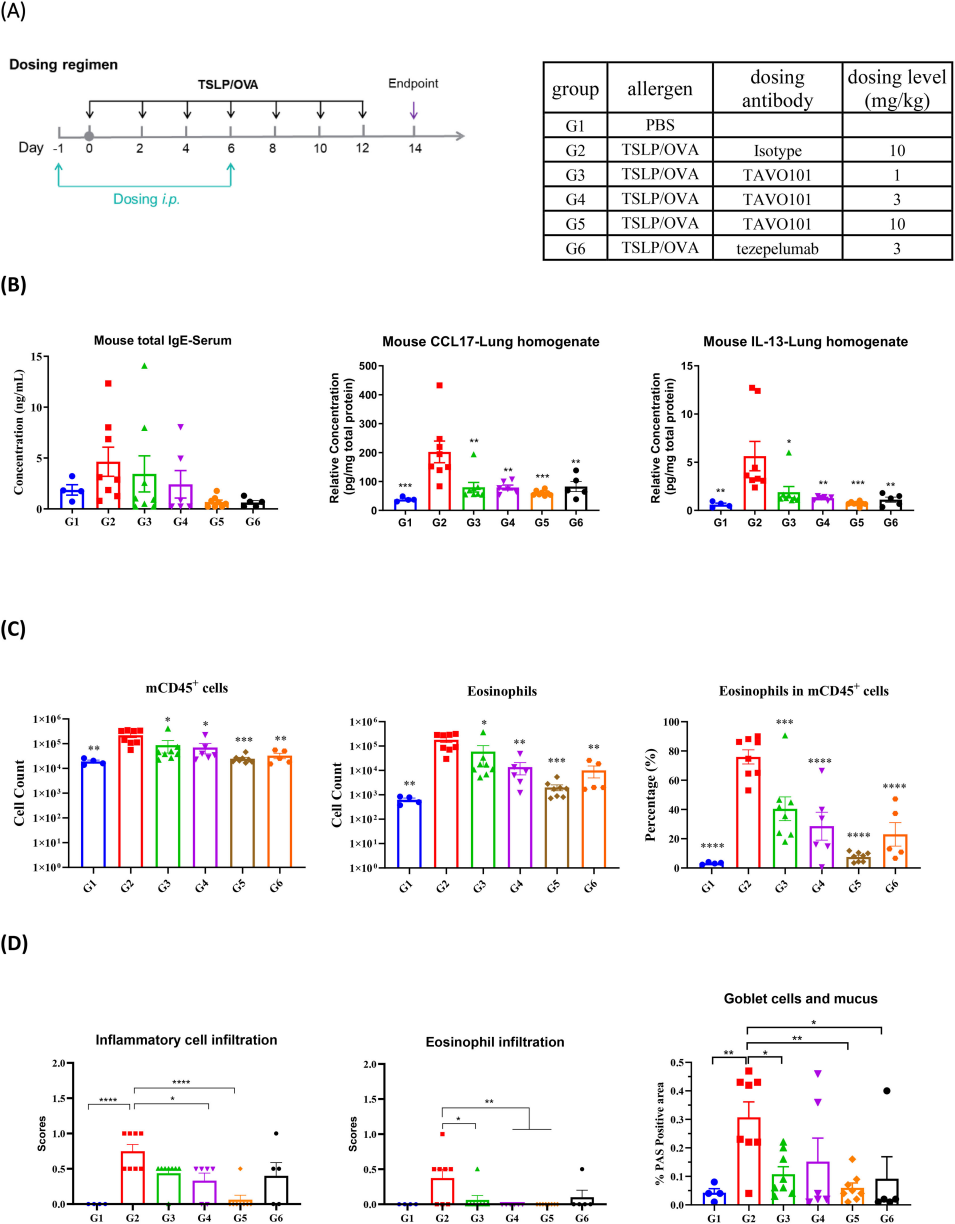
Efficacy of TAVO101 in TSLP/OVA-induced asthma model using hTSLP/hTSLPR humanized mice. **(A)**. Dosing regimen and animal grouping in the asthmatic model. Four mice were enrolled in the G1 group while eight mice were enrolled in each of the G2 to G6 groups. **(B)**. The concentration of mouse total serum IgE and lung tissue CCL17 and IL-13. **(C)**. Cell counts of mouse CD45^+^ leukocytes and eosinophils and the associated percentage of eosinophils in CD45^+^ leukocytes in BALF of asthmatic mice. **(D)**. Scores of inflammatory cell infiltration and eosinophil infiltration by H&E staining and the positive area proportion of Goblet cells and mucus of asthmatic lungs. Note: Data was represented by mean ± SEM and analyzed by One-way ANOVA with Dunnett’s multiple comparisons test. Comparison between each experimental group and G2 group. (*p<0.05, **p<0.01, ***p<0.001, ****p<0.0001).

Since TAVO101 did not recognize murine TSLP, the *in vivo* efficacy of TAVO101 was evaluated in hTSLP/hTSLPR humanized mice in which the endogenous mouse *TSLP* and *TSLPR* were genetically replaced by their corresponding human sequences. The allergic asthma disease state was induced by the administration 10 μg OVA (ovalbumin) and 1 μg human TSLP intranasally into these mice every other day for two weeks (**Figure 3A**). TAVO101 at doses of 1 mg/kg, 3 mg/kg, and 10 mg/kg were administered once a week via intraperitoneal injection on Days -1 and 6. Likewise, the isotype control antibody at a dose of 10 mg/kg and tezepelumab at a dose of 3 mg/kg were administered as controls with the same dosing scheme. The condition and body weight of the mice were monitored throughout the study. On day 14, serum, bronchoalveolar lavage fluid (BALF) samples and lung tissues were collected for ELISA, flow cytometry and lung histopathology analysis.

During the entire experimental period, all animals maintained normal food intake and the body weights of the animals in each experimental group gradually increased over time and reached to a similar level at the conclusion of the study. These animal profiles showed that the test articles were well tolerated (**Supplementary Figures 3A, B**).

Total IgE levels in serum and key cytokine levels in lung tissues were measured on day 14 to assess the efficacy of anti-TSLP antibodies in this asthma model. To confirm the successful establishment of the asthma model, mice in G2 group administered with TSLP/OVA showed significantly elevated serum IgE levels when compared to mice in G1 group without the TSLP/OVA challenge (**Figure 3B**). Administration of TAVO101 resulted in a substantial dose-dependent reduction in serum total IgE levels (G3-G5 groups) when compared to the G2 isotype control group. Likewise, tezepelumab dosed at 3 mg/kg also led to substantial reduction in serum total IgE to a level similar to that with 10 mg/kg TAVO101 dosing (**Figure 3B**). For key cytokine levels associated with asthma in the lung, TSLP/OVA administration led to significant increases of lung CCL17 and IL-13 levels in mice in G2 group relative to G1 group. The treatment with TAVO101 and tezepelumab significantly mitigated the increase of these two cytokines (**Figure 3B**). However, the addition of TSLP/OVA and anti-TSLP antibody did not change lung IL-4 and IL-5 levels (**Supplementary Figures 3C, D**).

Flow cytometry analysis of cells isolated from bronchoalveolar lavage fluid assessed the infiltration of inflammatory cells associated with asthma. A significant increase in the cell counts of CD45^+^ leukocytes and eosinophils and the associated percentage of eosinophils in CD45^+^ leukocytes were observed in the BALF of mice in the G2 group with the TSLP/OVA challenge when compared to the G1 group without the challenge (**Figure 3C**). Relative to the G2 isotype control group, treatment with TAVO101 dose-dependently reduced the number and proportion of CD45^+^ leukocytes and eosinophils in the BALF of mice of the G3 to G5 groups. Treatment with tezepelumab also led to similar significant reduction in CD45^+^ leukocytes and eosinophils in the G6 group. However, no significant changes in the cell counts of BALF neutrophils and macrophages were observed among different experimental groups (**Supplementary Figures 3E, F**).

The inflammatory cell infiltration and mucus production in the bronchioles were assessed by histopathological staining and scoring of the asthmatic animal lungs. By hematoxylin and eosin (H&E) staining, a significant increase of infiltration of inflammatory cells and eosinophils around blood vessels and bronchioles were observed in the lungs of G2 group of mice relative to the G1 control group, indicating the establishment of asthma-related lesions by TSLP/OVA administration (**Figure 3D**). Treatments with the anti-TSLP antibodies TAVO101 and tezepelumab significantly reduced the infiltrations of inflammatory cells and eosinophils. Besides, the Goblet cells and mucus in bronchioles were analyzed by Periodic Acid Schiff (PAS) staining. A significant increase of mucus overproduction and Goblet cell metaplasia were observed in the asthmatic lung of mice in G2 group upon TSLP/OVA stimulation, while such disease phenotypes were significantly mitigated by the treatment of TAVO101 and tezepelumab in the lungs of G3 to G6 groups of mice (**Figure 3D**). These data from the murine asthma model exhibited how TAVO101 could mitigate different markers of asthma.

### Efficacy of TAVO101 in an imiquimod induced psoriasis mouse model

**Figure 4:**
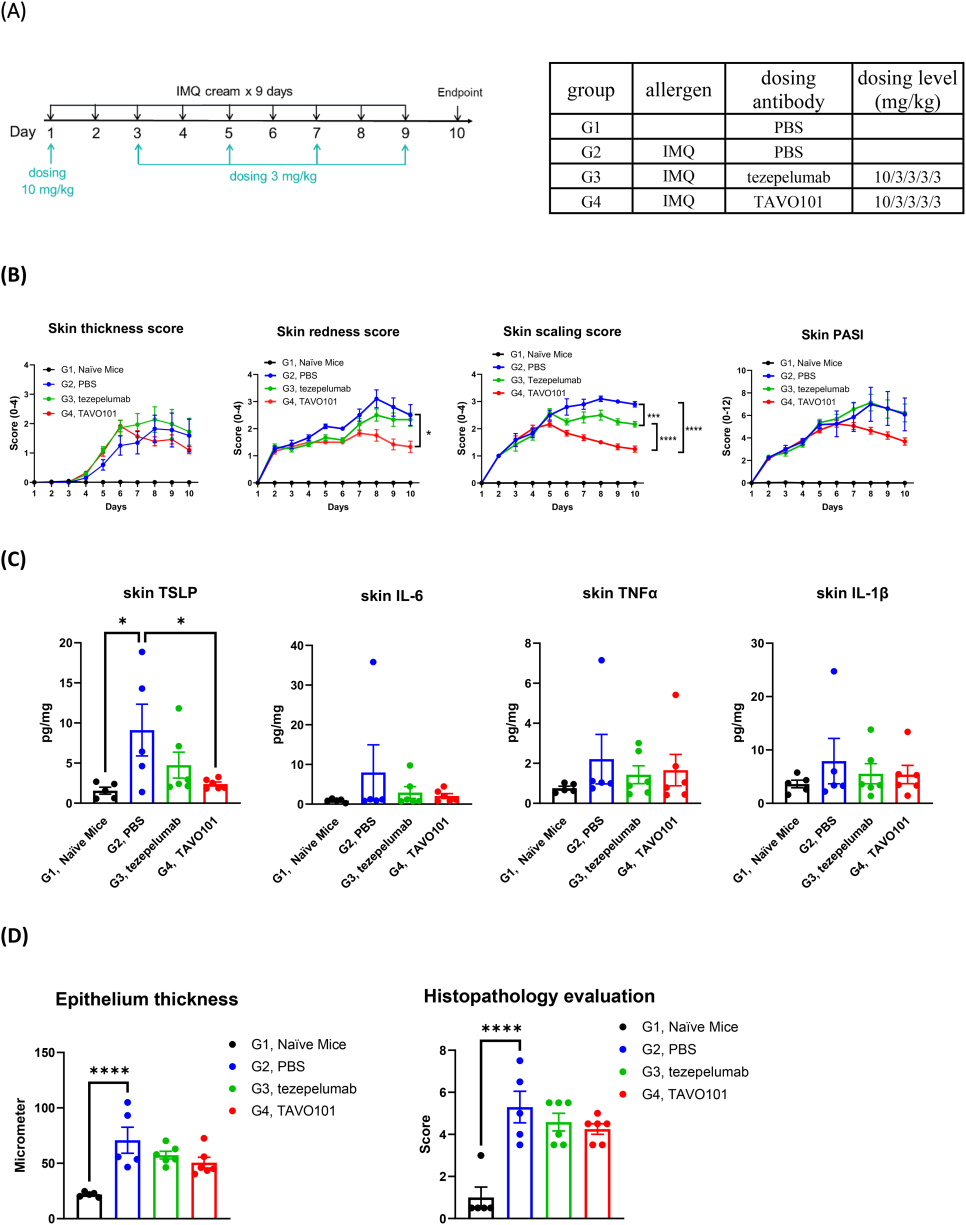
Efficacy of TAVO101 in an imiquimod induced psoriasis mouse model using hTSLP/hTSLPR humanized mice. **(A)**. Dosing regimen and animal grouping in the psoriasis model. Five mice were enrolled in the G1 group while six mice were enrolled in each of the G2, G3 and G4 groups. **(B)**. Skin lesion thickness, redness, scaling and PASI score changes of each treatment group throughout the study. **(C)**. Levels of human TSLP, mouse IL-6, mouse TNFα and mouse IL-1β in skin lesions of each treatment group at the study end. **(D)**. The epithelium thickness and histopathological score of skin lesions of each treatment group at the study end. Data was represented by mean ± SEM and analyzed by One-way ANOVA with Tukey’s or Dunnett’s multiple comparisons test. Statistical analyses were performed on Day 10 data in Figure B and on comparison between each experimental group and G2 group in Figures C and D (*p<0.05, **p<0.01, ***p<0.001, ****p<0.0001).

The *in vivo* efficacy of TAVO101 was also evaluated in an imiquimod (IMQ) induced psoriasis mouse model using hTSLP/hTSLPR double knock-in mice. The skin psoriasis was induced and established by topical application of 62.5 mg/mouse Aldara cream containing 5% IMQ on the exposed back skin of mice every day, while Vaseline was applied on exposed skin of naïve mice in control group (**Figure 4A**). Following IMQ application, TAVO101 or tezepelumab were administered to the mice via intraperitoneal injection with the dose at 10 mg/kg on Day 1 followed by four additional doses at 3 mg/kg every other day (10/3/3/3/3 dosing). The skin thickness, redness and scaling were measured and scored every day. At the end of study, the skin lesion samples were collected for histopathological analysis and the quantitation of key inflammatory cytokine levels.

Upon the application of Aldara cream containing 5% IMQ on the exposed back skin, mice in groups G2 to G4 experienced decreases in body weight during the first two days then gradually recovered back to normal by Day 8 (**Supplementary Figure 4A**). Besides, at the study end there were significant increase in spleen weights for groups G2 to G4 mice with the 5% IMQ application relative to G1 group mice without such application (**Supplementary Figure 4B**). Notably, mice in groups G3 and G4 which were treated with tezepelumab and TAVO101 respectively showed similar degrees of body weight drops and increases in spleen weight compared to group G2 mice without the antibody treatment, indicating that the anti-TSLP antibodies were well tolerated by the animals.

Compared with control mice in group G1 receiving Vaseline application, mice in groups G2 to G4 showed a statistically significant induction in clinical skin inflammation features including skin thickness, skin redness, skin scaling, and skin PASI (Psoriasis Area and Severity Index) throughout the study (**Figure 4B** and **Supplementary Figure 4C**), indicating the successful induction of mice psoriasis with multiple repeated IMQ treatment. Treatment with either TAVO101 in G4 group or tezepelumab in G3 group led to anti-inflammatory effects including decreased skin thickness and reduced skin redness, scaling, and PASI scores when compared to the G2 group without antibody treatment. Particularly, TAVO101 administration exhibited a trend of better treatment effects than tezepelumab in every clinical skin inflammation measurement, although statistically significant difference was achieved only for the skin scaling score on Day 10 (**Figure 4B** and **Supplementary Figure 4C**).

Key inflammatory cytokine levels in the skin lesions were determined at the end of the study. Topical application of IMQ on the exposed skin in G2 group mice induced significantly elevated human TSLP secretions compared to G1 group mice without IMQ application (**Figure 4C**). Either TAVO101 treatment in G4 group or tezepelumab treatment in G3 group significantly mitigated the secretion of human TSLP in skin lesions. Among key inflammatory cytokines, substantial increases in IL-6, TNFα and IL-1β levels in the skin lesions were observed upon IMQ stimulation, while such induction of these inflammatory cytokines were mitigated by the treatments of either TAVO101 or tezepelumab (**Figure 4C**).

The skin lesions were also subjected to pathological analysis for the measurement of epithelium thickness and the scoring of degree of skin histopathological lesion. Representative pictures of skin histopathological lesion of each group were exhibited in **Supplementary Figure 4D**. IMQ application led to significant increase in epithelium thickness and skin histopathological lesion scores in the skin lesions of G2 group of mice (**Figure 4D**). Treatment with either TAVO101 or tezepelumab showed some therapeutical effects in terms of decreased epithelium thickness and alleviated skin histopathological lesion scores. In summary, TAVO101 also showed significant efficacy in this imiquimod induced psoriasis mouse model.

### Fc engineering of TAVO101 antibody for half-life extension and reduced effector functions

The fragment crystallizable region (Fc region) of IgG antibody can be engineered to modulate Fc receptor engagement to obtain desired therapeutic and pharmacokinetic profile. To further develop TAVO101 antibody as a therapeutic biologics drug for allergic diseases, Fc engineering technologies were adopted to improve pharmacokinetic profile with reduced potentials of Fc-mediated cytotoxicity of TAVO101 antibody. Specifically, M428L/N434S mutations have been demonstrated to extend antibody half-life by increasing FcRn binding affinity (36). L234A and L235A double mutations in the lower hinge region have been shown to abolish the effector functions of IgG1 antibody by reducing its binding to Fcγ receptors (37). Therefore, a set of four mutations, L234A, L235A, M428L, N434S, collectively designated as AALS mutations, were introduced into the IgG1 Fc of TAVO101 antibody to make the TAVO101_IgG1-AALS antibody.

**Figure 5:**
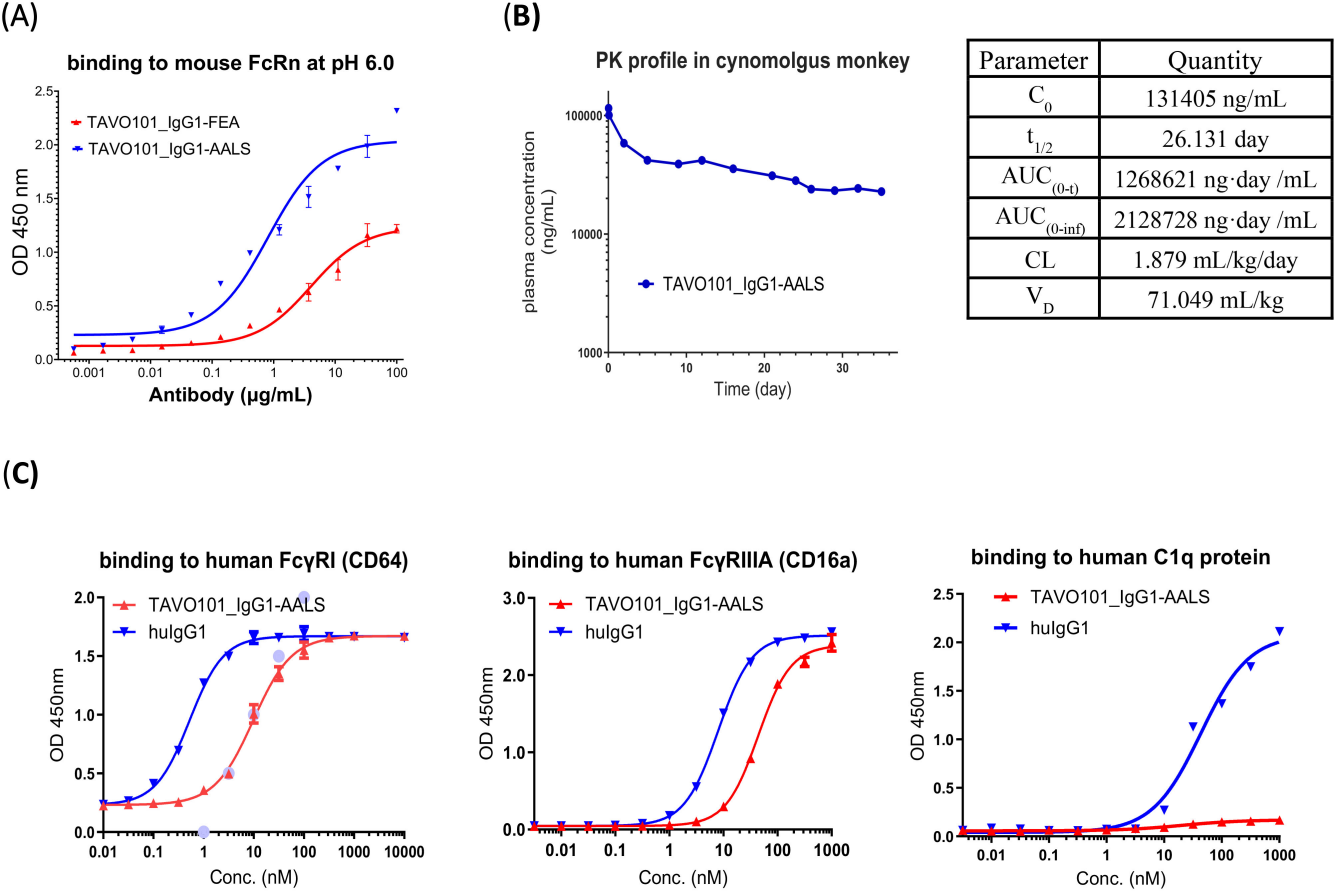
Fc engineering of TAVO101 for extended half-life and reduced Fcγ receptor binding. **(A)**. Binding to mouse FcRn by Fc engineered TAVO101. Increasing concentrations of TAVO101_IgG1-AALS and TAVO101_IgG1-FEA were assessed for their binding to immobilized mouse FcRn at pH 6.0 in ELISA assays. OD at 450 nm were plotted against the concentrations of test antibodies (Data expressed as mean ± SEM, n=2). **(B)**. Pharmacokinetic profile of an Fc engineered TAVO101. TAVO101_IgG1-AALS antibody was administered as a single 4 mg/kg intravenous dose into a cynomolgus monkey. The antibody concentrations in plasma at time points up to day 35 post-dose were quantitated and plotted against the testing days. The calculated pharmacokinetic parameters were shown. **(C)**. Binding to human Fcγ receptors and C1q complement protein by Fc engineered TAVO101. Increasing concentrations of TAVO101_IgG1-AALS antibody and a human IgG1 control antibody were assessed for their binding to FcγRI (CD64), FcγRIIIA (CD16a), and C1q. OD at 450 nm were plotted against the concentrations of test antibodies (Data expressed as mean ± SEM, n=3).

Whether the Fc-engineered antibodies have improved FcRn binding affinity were assessed in an ELISA-based binding assay. Incremental concentrations of TAVO101_IgG1-AALS antibody were applied to ELISA plate for their binding to coated mouse FcRn under pH 6.0. As a comparison, TAVO101_IgG1-FEA, a TAVO101 antibody with the L234F, L235E and D265A (FEA) Fc mutations which abrogate the effector functions similarly as L234A and L235A double mutations (38), but without the half-life extension M428L/N434S mutations, was also assessed. It was observed that TAVO101_IgG1-AALS antibody bound FcRn with better potency and efficacy than TAVO101_IgG1-FEA antibody, owing to the presence of M428L and N434S mutations intended for half-life extension (**Figure 5A**).

To determine whether the M428L/N434S mutations could extend the circulating half-life of Fc-engineered anti-TSLP antibody, a pharmacokinetic (PK) study was performed in a cynomolgus monkey. TAVO101_IgG1-AALS antibody was administered as an intravenous infusion. There were no clinical safety findings (e.g., body weight loss, itching, dyspnea, anorexia) observed throughout the entire 5 weeks study period. The PK data were analyzed for half-life, C_0_, AUC, V_D_, and clearance (CL) and the TAVO101_IgG1-AALS antibody concentrations in plasma were plotted as a function of time (**Figure 5B**). TAVO101_IgG1-AALS antibody plasma concentrations showed expected biphasic pharmacokinetics with a rapid alpha phase followed by a slower linear terminal elimination (beta) phase, with an approximate calculated half-life of 26 days, which is 2-3 fold extension of a typical human IgG based therapeutics in the monkey. This data revealed a significant improvement in circulating half-life for TAVO101_IgG1-AALS antibody with the engineered mutations intended for the extension.

To assess whether Fc-engineered antibodies may have reduced interactions to Fcγ receptors, the binding to human FcγRIIIA (CD16a) and FcγRI (CD64), which are the major Fcγ receptors mediating antibody-dependent cellular cytotoxicity (ADCC) and antibody-dependent cellular phagocytosis (ADCP) activities, by TAVO101_IgG1-AALS antibody was assessed in ELISA-based binding assays. TAVO101_IgG1-AALS antibody showed nearly 5-fold and 20-fold reduced potency in binding to FcγRIIIA (CD16a) and FcγRI (CD64), respectively, relative to a control human IgG1 antibody without the engineered L234A and L235A Fc mutations (**Figure 5C**). Besides, a similar ELISA-based binding assay was performed to evaluate TAVO101_IgG1-AALS antibody binding to human complement component C1q protein which is a subunit of the C1 enzyme complex that mediates Complement-Dependent Cytotoxicity (CDC). TAVO101_IgG1-AALS antibody showed negligible binding to C1q, while the control human IgG1 antibody without the engineered L234A and L235A Fc mutations showed strong binding (**Figure 5C**). In summary, TAVO101 Fc domain was engineered to extend half-life by having higher binding affinity to FcRn and reduced binding to major Fcγ receptors and complement C1q component.

## Discussion

While TSLP plays a pivotal role in allergic inflammation, the excess activity of this pro-inflammation cytokine implicates the initiation and progression of many different types of allergic, chronic inflammation and autoimmune diseases (8). We identified a humanized anti-TSLP antibody TAVO101 with high binding affinity to human TSLP and blockade TSLP binding to its cell surface receptor complexes. TAVO101 showed potent neutralization of TSLP activities in several *in vitro* cell-based functional assays. TSLP exerts its cellular effects by binding to and phosphorylation of TSLPR and then IL-7Rα receptor to form an active ternary signaling complex. The activated receptors recruits and phosphorylates JAK2 and then induces the activation of multiple STAT proteins represented by STAT5 to drive target gene expression critical for allergic inflammation (11). This TSLP-driven signaling cascade is recapitulated in the STAT5 reporter assay, in which the binding of TSLP to its receptor complexes triggers a signaling cascade leading to the activation of STAT5 and the subsequent activation of reporter gene which is under the control of a promoter with five STAT5 binding sites. The potent neutralization of reporter gene activation by the TAVO101 antibody in the STAT5 reporter assay indicated that the antibody effectively blocked TSLP activity in this signaling pathway. Besides JAK-STAT signaling pathway, TSLP can also drive cellular proliferation independent of STAT5 activation, mediated by Src-type of mediators (14). In this regard, we developed a cell proliferation assay in which TSLP can drive the proliferation of pro-B cell line BaF3 with expression of human TSLPR and IL-7Rα receptors. The TAVO101 antibody also neutralized TSLP activity in driving BaF3 cell proliferation. In summary, the cell-based functional assays demonstrated that the TAVO101 antibody could neutralize both mechanisms of action of TSLP in activating target gene expression and cell proliferation critical for allergic inflammation responses.

As a proinflammatory cytokine, TSLP can activate its target cells, mainly dendritic cells, to release inflammatory cytokines and chemokines to promote naïve CD4^+^ T cells to adopt a Th2 phenotype (39). As an *ex vivo* model for this cellular response, we developed a CCL17 release assay in which TSLP can stimulate the activation of primary human CD1c^+^ dendritic cells with increased secretion of CCL17, a chemokine critical for mediating Th2 type allergic inflammation response (34). TAVO101 blocked TSLP-driven CCL17 release from activated dendritic cells isolated from two different PBMC donors. Besides activating dendritic cells, TSLP can also directly stimulate the proliferation of human CD4^+^ T cells when the T cells are pre-activated by TCR engagement that leads to the expression of TSLP receptors (35). We observed that TSLP could augment the proliferation of CD4^+^ T cells activated by coated anti-CD3 antibody. TAVO101 showed a trend of slightly better efficacy (albeit not statistically significant) than tezepelumab in blocking this TSLP-mediated proliferation of T cells isolated from two PBMC donors. These *ex vivo* studies demonstrated that the TAVO101 inhibited TSLP-driven allergic inflammation responses from its target cells which laid the foundation for its potential as a therapeutic treatment of TSLP-mediated allergic, chronic inflammatory and autoimmune diseases.

The lack of cross-reactivity to murine TSLP hindered the assessment of tezepelumab and TAVO101 efficacies in TSLP-driven diseases by mouse models. To overcome this hurdle, humanized mice with transgenic of both human *TSLP* and human *TSLPR* to replace native murine counterparts were generated. The capability of induction of typical asthma phenotypes, including airway hyperresponsiveness, mucus production, eosinophilic infiltration and elevated IgE level, by the intranasal administration of human TSLP along with OVA corroborated the functional integrity of human TSLP-TSLPR signal cascade in the transgenic mice. In imiquimod induced psoriasis mouse model, the induction of human TSLP expression in the skin lesions upon IMQ application confirmed the equivalent temporal and spatial pathophysiological expression of human *TSLP* transgene as its murine counterpart. Besides, the significant mitigation of imiquimod induced psoriasis by anti-human TSLP antibodies TAVO101 and tezepelumab also validated the substantial contribution of a functional human TSLP-TSLPR signaling cascade in the establishment of IMQ induced psoriasis in the transgenic mice.

In TSLP/OVA induced asthma model, the intranasal administration of TSLP/OVA significantly induced the elevation of serum IgE level and key inflammatory cytokines CCL17 and IL-13 levels in the lung which contributed to the disease establishment and progression. However, there were no significant changes in IL-4 and IL-5 levels in the lung at the end of the study. Both TAVO101 and tezepelumab showed similar efficacy in blocking the induction of serum IgE levels and CCL17 and IL-13 levels in the asthmatic lung. The *in vivo* effect on CCL17 correlated with our *ex vivo* study of TAVO101 in blocking TSLP-driven CCL17 release from activated CD1c^+^ dendritic cells. At the cellular level, TSLP/OVA induced asthma was accompanied with a significant infiltration of CD45^+^ leukocytes and eosinophils which are crucial in allergic responses during asthma pathogenesis, but not neutrophils or macrophages, in the BALF of asthmatic mice. TAVO101 dose-dependently reduced the percentage of eosinophil in CD45^+^ leukocytes to a baseline level close to that without TSLP/OVA induction. As a result of the blockade of key molecular and cellular contributors of asthmatic pathogenesis, TAVO101 significantly mitigated the mucus overproduction and Goblet cell metaplasia in the asthmatic lung as revealed by histopathological analysis.

As a ligand for Toll-like receptors 7/8, imiquimod induced the release of TSLP in the skin lesions as a critical mediator of the pathogenesis of IMQ induced psoriasis. This is evidenced by the effectiveness of anti-human TSLP antibodies in the mitigation of skin psoriasis hallmarks, including reduced skin thickness, erythema, scaling, epidermal hyperplasia and reduced histopathological skin lesions such as hyperkeratosis, acanthosis, and parakeratosis. TAVO101 exhibited a trend of better efficacies than tezepelumab in reducing skin lesions in the psoriasis model, likely owing to the slightly more potent neutralization of skin TSLP than tezepelumab dosed at the same levels. Mechanistically, blocking TSLP by anti-TSLP antibodies reduced the elevation of key inflammatory cytokines, including IL-6, TNFα and IL-1β, and the increased infiltration of inflammatory cells within the skin lesions.

At the molecular level, TAVO101 exerts its neutralization activity by blocking TSLP binding to its cell surface receptor complexes as revealed by flow cytometry based competitive binding assay. Although TAVO101 showed binding affinity to TSLP comparable to tezepelumab in both ELISA and flow cytometry-based assays, BLI based competition binding assay revealed that TAVO101 antibody binds to TSLP on an epitope distinct from that for tezepelumab. The differential binding properties of TAVO101 and tezepelumab on cynomolgus monkey TSLP also confirmed their differences in binding epitopes. Further functional assays revealed that the TAVO101 is over 10-fold more potent than tezepelumab in the neutralization of TSLP-driven signal cascade mediated by STAT5. In addition, TAVO101 showed a trend of slightly more efficacious than tezepelumab in inhibiting TSLP-driven proliferation of activated CD4^+^ T cells in an *ex vivo* study and in an *in vivo* study with imiquimod induced psoriasis mouse model. Therefore, although TAVO101 binds to TSLP with similar affinity relative to tezepelumab, binding to a distinct epitope might augment its effectiveness of blocking TSLP-mediated receptor activation, which leads TAVO101 to have a more efficient neutralization of TSLP activities than tezepelumab.

To develop humanized TAVO101 antibody as a drug molecule, we introduced L234A and L235A mutations in its IgG1 Fc. This optimized antibody showed reduced binding affinity to Fcγ receptors which are expressed in many tissues, especially on the surface of cells of the immune system and liver Kupfer cells. The reduced FcγR binding and associated effector functions may facilitate Fc-engineered TAVO101 with improved drug biodistribution with reduced non-specific liver accumulation and a more favorable safety profile with minimal unwanted inflammatory responses (40, 41). Besides, we also engineered M428L and N434S Fc mutations in the TAVO101 antibody (36). For experimental convenience, we used the mouse FcRn to confirm the levels of Fc-FcRn engagement. Mouse FcRn is known binding to human Fc with higher affinity than human FcRn. As expected, such optimized antibody also showed increased affinity to FcRn receptor and thus much longer half-life in the preclinical monkey PK model. With such optimization, less frequent dosing can be achieved for the TAVO101 which will facilitate patient convenience and compliance and help to reduce the generation of anti-drug antibody. More importantly, allergens attack under unpredictable time and conditions. A prolonged TSLP blocking under these disease conditions facilitated by engineered TAVO101 may become critical for effective control of the flaring and exacerbation.

As the only TSLP antagonist developed to clinical stage, tezepelumab has been approved for the treatment of severe asthma and is being evaluated in multiple clinical trials for its efficacy in additional TSLP-mediated diseases, including severe chronic rhinosinusitis with nasal polyposis, chronic obstructive pulmonary disease, and chronic spontaneous urticaria. With similar or better potencies in the neutralization of TSLP activities, TAVO101 can be efficacious in the treatment of all these TSLP-mediated disorders. Besides the asthma and psoriasis mouse models, TAVO101 also showed a good efficacy in a murine atopic dermatitis model (33). Nonetheless, we will examine how the efficacies in animal models can be translated to clinical benefits in our planned clinical trials for multiple TSLP-mediated allergic diseases. Besides, the extended half-life of TAVO101 with reduced Fc-mediated immune cell engagement could expand its clinical utility. Indeed, our recent Phase 1 clinical trial indicated that TAVO101 was safe, well-tolerated and exhibited a median half-life of 67 days with no observation of anti-drug antibody development (33), although the long-term effects of TAVO101 in clinical setting remain to be explored.

Recent studies highlighted that dual blockade of key mediators with distinct or overlapping roles in inflammation may provide improved synergistic efficacy in disease control over single blockade (42–44). TAVO101 antibody, beside further development in the clinic, created a fundamental building block for additional discovery of bispecific antibodies or in combination with antibodies to other critical inflammatory mediators as therapeutics to a broader spectrum of allergic, inflammation and autoimmune diseases. In summary, TAVO101 has the potential to be developed as the best-in-class biologics drug for the therapeutic of TSLP-mediated diseases.

## Data Availability

The original contributions presented in the study are included in the article/**Supplementary Material**. Further inquiries can be directed to the corresponding author.
