# Supplementary material for: A novel monoclonal antibody against human thymic stromal lymphopoietin for the treatment of TSLP-mediated diseases
Source: Front Immunol. 2024 Dec 11;15:1442588. doi: 10.3389/fimmu.2024.1442588 (PMC11670205; doi:10.3389/fimmu.2024.1442588)
Supplement: Supplementary file 1 [file DataSheet1.docx]

Supplemental Figure 1

**A** **B**

**Supplemental Figure 1**. TSLP binding for TAVO101 and tezepelumab. Increasing concentrations of TAVO101 and tezepelumab were assessed for their binding to immobilized cynomolgus monkey TSLP (**A**) or mouse TSLP (**B**). OD at 450 nm were plotted against the concentrations of test antibodies (Data expressed as mean ± SEM, n=2).

Supplemental Figure 2

**A** **B**

**C**


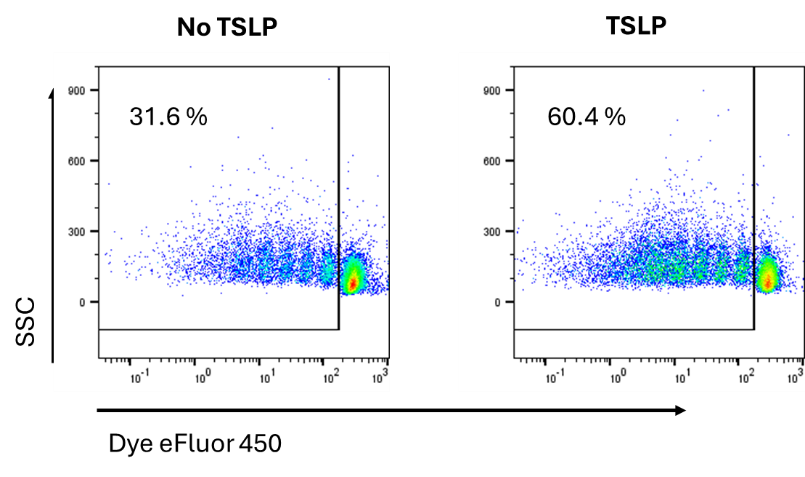


**Supplemental Figure 2**. TSLP activities in functional potency assays. (**A**). TSLP-driven STAT5 reporter gene activation assay. Increasing amounts of human TSLP were applied to HEK293T cells transfected with human TSLP receptor complex and a STAT5-responsive luciferase reporter gene and reporter gene expression was quantitated. The Relative Light Units (RLU) were plotted against the concentrations of TSLP (Data expressed as mean ± SEM, n=2). (**B**). TSLP-driven proliferation of BaF3 cells transfected with human TSLP receptor complex. Increasing amounts of human TSLP were applied to the transfected cells and cell proliferation was quantitated. Luminescence signals reflecting cell proliferation were plotted against the concentrations of TSLP (Data expressed as mean ± SEM, n=3). (**C**). TSLP-driven proliferation of human CD4^+^ T cells. Human CD4^+^ T cells were labelled by Cell Proliferation Dye eFluor 450 and activated for six days with plate bound anti-CD3 antibody with or without 50 ng/mL TSLP. Dye eFluor450 dilution in CD4^+^ T cells were analyzed by flow cytometry and plotted. The fraction of proliferated T cells with diluted eFluor 450 signals were boxed and quantitated.

Supplemental Figure 3

**A** **B**

**C D**

**E F**

**Supplemental Figure 3**. Efficacy of TAVO101 in an TSLP/OVA-induced asthma model using hTSLP/hTSLPR humanized mice. (**A**) Mouse body weight changes for each group over the study period. (**B**) Day 14 mouse body weight comparisons between groups. (**C**). The concentration of mouse lung tissue IL-4 in each group. (**D**). The concentration of mouse lung tissue IL-5 in each group. (**E**). Cell counts of mouse neutrophils in BALF of asthmatic mice. (**F**). Cell counts of mouse macrophages in BALF of asthmatic mice. Data was represented by mean ± SEM.

Supplemental Figure 4

**A B C**

 ****

**D**


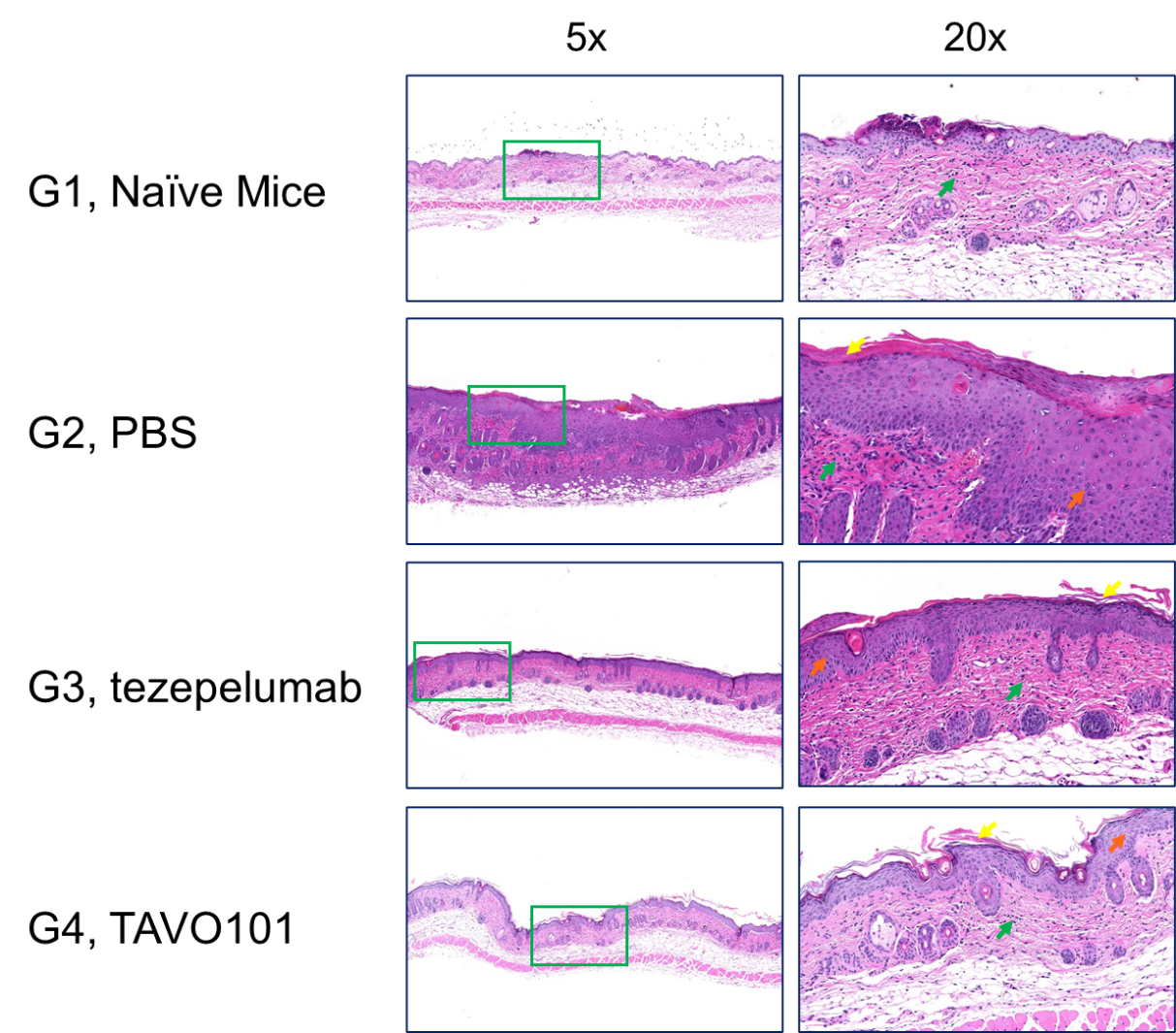


**Supplemental Figure 4**. Efficacy of TAVO101 in an imiquimod induced psoriasis mouse model using hTSLP/hTSLPR humanized mice. (**A**) Mouse body weight changes for each group over the study period. (**B**) Day 10 mouse spleen weight comparisons between groups. Data was represented by mean ± SEM and analyzed by One-way ANOVA with Dunnett's multiple comparisons test. Comparison between each experimental group and G2 group. (*p<0.05, ** p<0.01, *** p<0.001, **** p<0.0001). (**C**). Skin thickness changes of each treatment group throughout the study. (**D**). Representative pictures (5x image and 20x highlight of inset) of histopathological analysis of skin lesions by H&E staining for each group. Hyperkeratosis was shown as yellow arrow. Acanthosis was shown as orange arrow. Inflammatory cell infiltration was shown as green arrow.

Supplemental Table 1

| Inflammatory cell infiltration around blood vessels and bronchioles | |
| --- | --- |
| a. the infiltration area accounted for less than 5% of the total area | 0.5 |
| b. the infiltration area accounted for 5% to 20% of the total area | slight 1.0  moderate 1.5  severe 2.0 |
| c. the infiltration area accounted for 20% to 40% of the total area | slight 2.5  moderate 3.0  severe 3.5 |
| d. the infiltration area accounted for more than 40% of the total area | 4.0 |
| Eosinophil infiltration | |
| a. minimal | 0.5 |
| b. slight | 1.0 |
| c. moderate | 1.5 |
| d. severe | 2.0 |
